# STED/AFM as a tool to investigate mechanical and adhesive properties of migrating keratinocytes

**DOI:** 10.1080/19336918.2026.2657675

**Published:** 2026-04-15

**Authors:** Mariya Y. Radeva, Jens Waschke, Michael Fuchs

**Affiliations:** Vegetative Anatomy, Institute of Anatomy, Faculty of Medicine, LMU Munich, Munich, Germany

**Keywords:** E-Cadherin, keratinocyte migration, STED/AFM, force spectroscopy, adherens junctions

## Abstract

E-cadherin is a key component of adherens junctions which maintains epithelial integrity. In keratinocytes, wound healing requires dynamic modulation of adhesion and cytoskeletal organization. Using a wound healing assay combined with stimulated emission depletion/atomic force microscopy (STED/AFM), we analysed E-cadherin binding during murine keratinocyte migration. Wound closure occurred within 6 h and was accompanied by E-cadherin accumulation at the leading edge. Transient expression of E-Cadherin-SNAP enabled investigation of E-cadherin interactions. Inhibition of actin polymerization abolished E-cadherin binding and reduced cellular stiffness. Imaging of SiR-actin-labeled cells enabled simultaneous visualization of migration and measurement of binding and mechanical properties. E-cadherin retained functional binding properties during migration. These findings establish STED/AFM as a powerful method to investigate single-molecule binding properties in migrating cells.

## Introduction

E-Cadherin (E-Cad) is a transmembrane glycoprotein that mediates calcium-dependent intercellular adhesion and is a core component of the adherens junction (AJ) complex [1]. The primary function of AJs is to maintain intercellular adhesion and provide resistance to external mechanical forces. E-Cad connects to the actin cytoskeleton via intracellular plaque proteins [2,3]. Cell–cell adhesion mediated by AJs is a highly dynamic process, playing a crucial role during epithelial-to-mesenchymal transition (EMT), both during normal development and along carcinogenesis [3,4]. In adult tissues, the loss of E-Cad is a key event in epithelial tumorigenesis and is observed across a wide range of tumor types. Conversely, re-expression of E-Cad in cancer cells has been shown to prevent tumor progression [5,6]. In cancer, reduced E-Cad expression is a hallmark of the invasive cell phenotype [7].

Skin wound healing is a highly intricate process that typically progresses through three overlapping phases: inflammation, tissue formation, and remodeling [8,9]. During the tissue formation phase, in addition to angiogenesis and extracellular matrix deposition, keratinocytes migrate across the fibrin matrix initially settled at the base of the wound to reestablish the epidermal barrier, an essential step in wound closure both *in vitro* and *in vivo* [10]. At the wound edge, keratinocytes undergo a partial reversible EMT, also termed epithelial–mesenchymal plasticity (EMP), enabling them to regain migratory capacity [11]. In this state, E-Cad expression is downregulated through activation of the epidermal growth factor (EGF) receptor, which in turn reduces intercellular adhesion and facilitates keratinocytes to migrate across the wound bed [11,12]. Interestingly, E-Cad is not completely lost during this process. Instead, the remaining E-Cad molecules sustain a degree of cell–cell adhesion, supporting a mechanism known as collective migration [13]. However, in migrating keratinocytes, the binding properties and functional role of the leftover E-Cad molecules remain poorly understood.

To investigate both the mechanical and the binding properties of E-Cad in migrating murine keratinocytes (MEK), we performed scratch wound assays, immunostaining analysis and a novel hybrid approach that combines STED microscopy with AFM single-molecule force spectroscopy (SMFS) [14,15]. Using this STED/AFM system, we were able to measure specific E-Cad adhesion events on living, migrating MEK.

Our results show that E-cad molecules present during migration retain binding properties comparable to those in stable adherens junctions, indicating they remain functional and available for rapid re-engagement in cell–cell adhesion. A preliminary version of this article has been made available as a preprint [16].

## Materials and methods

### Cell culture, transfection, and reagents

Wild-type MEK were isolated and cultured as previously described [17]. Cells were maintained in a complete FAD medium (ThermoFisher Scientific, Germany) and initially grown in a low-calcium medium (0.05 mM CaCl_2_). Upon reaching confluency, the culturing conditions were switched, and the cells were cultivated under high-calcium conditions (1.8 mM CaCl_2_) to induce differentiation. All experiments were conducted 24 h after the calcium switch was initiated. For E-Cad overexpression, MEKs were grown to 70% confluency and transiently transfected with the pSNAPf-ECad-N plasmid using Lipofectamine 3000, following the manufacturer’s instructions (Invitrogen, Carlsbad, USA) [18]. A day after transfection, the cells were exposed to high-calcium medium for another 24 h and then subjected to experiments. Inhibition of actin polymerization was achieved by 1 h pharmacological treatment with 2.0 µg/ml Latrunculin B (LatB; Merck, Darmstadt, Germany), as previously published [14].

### Wound assays

MEK cells were seeded into 24-well plates, with or without coverslips. After reaching confluency, cells were switched to high-calcium medium (1.8 mM CaCl_2_). Twenty-four hours later a scratch was introduced into the cell monolayer using a sterile P200 pipette tip. Cell migration was monitored over time using bright-field microscopy (Axio Vert A1; Carl Zeiss AG, Oberkochen, Germany) or immunostaining was performed at 0, 2, 4, 6, and 8 h post-scratch. For STED/AFM experiments, a sterile culture-Insert 2 well (Ibidi, Gräfelfing, Germany) was applied on the coverslip and removed 6 h before the test performance, to create a well-defined reproducible wound edge with little cellular debris for wound healing assays. This was done instead of a manual scratch to generate a cell-free gap.

### Immunostaining, labeling of actin filaments and cell nuclei

MEK cells were fixed by incubation with ice-cold absolute ethanol for 30 min, followed by a 3-min treatment with ice-cold acetone, both on ice. For detection of E-cad, a primary antibody directed against the protein was used (Invitrogen, Frankfurt, Germany). Actin filaments were visualized by Alexa 488-phalloidin (Dianova, Hamburg, Germany) and cell nuclei were stained by DAPI (Roche, Mannheim, Germany). For confocal microscopy, a Leica SP5 confocal microscopy with a 63 ×NA 1.4 PL APO objective controlled by LAS AF software (Leica, Mannheim, Germany) was utilized. For E-Cad intensity values, we used the whole region of interest and measured the integrated density values.

### Sample preparation for STED/AFM measurements

Staining of differentiated MEK’s monolayers was performed according to the manufacturer’s instructions. Briefly, cells exposed to complete high-calcium medium were incubated for 1 h with SiR-Actin (1 µM; Spirochrome, Denver, USA). After staining, the cells were washed once more with medium and transferred into the BioCell holder (Bruker Nano, Berlin, Germany), pre-heated to 37°C, within the integrated STED/AFM setup.

### Stimulated emission depletion microscopy (STED)

Imaging was performed using the Expert Line STED microscope system from Abberior (Abberior Instruments GmbH, Göttingen, Germany), equipped with a 100 × oil immersion objective. Image acquisition was carried out using the Imspector software (Abberior). Where applicable, fluorescent dye SNAP-CELL-TMR STAR was excited at 554 nm, whereas Sir-actin was excited at 652 nm, using pulsed diode lasers (PiL063X, Advanced Laser Diode Systems). STED depletion was achieved with a pulsed 775 nm laser operated at 10% to 30% power, with a gating delay of 800 ps. Fluorescence emission was detected using an avalanche photodiode detector within the spectral ranges 650–720 nm.

### Atomic force microscopy (AFM)

AFM measurements were performed exclusively within the STED/AFM setup using a NanoWizard 4 AFM system (Bruker Nano, Berlin, Germany). Cantilever functionalization followed previously well-established protocols [19–21]. The recombinant protein was applied at 0.15 mg/ml concentration and covalently linked to a flexible Si_3_N_4_ cantilever (MLCT probes, nominal spring constant 0.03 N/m, tip radius 20 nm; Bruker, Mannheim, Germany) via a heterobifunctional benzaldehyde polyethylene glycol (PEG) linker (Broadpharm, San Diego, USA). Tip calibration was performed using the contact-based method within the SPM software. Sensitivity was determined by recording force–distance curves on a rigid surface submerged in an aqueous environment. Linear fitting of the repulsive region allowed conversion of voltage signals to displacement units. The spring constant of the AFM tip was calculated from thermal noise measurements. Topographical imaging of MEK cells was conducted in Quantitative Imaging (QI) mode with the following settings: setpoint = 0.5 nN, Z-length = 1.5 µm, and scan speed = 50 µm/s. For live imaging in the QI mode, we used topography to obtain accurate height images and slope to visualize variations in sample stiffness. Adhesion measurements were performed using force mapping mode controlled by SPM Control v.4 software (JPK Instruments, Berlin, Germany) with parameters: relative setpoint = 0.5 nN, Z-length = 1.5 µm, extend/retract speed = 10 µm/s, and contact time = 0.1 s. Regions of interest were selected along cell–cell contact, surface, and leading edge. The scanned areas for force spectroscopy measurements cover 5 × 5 µm (25 ×25 pixels). Force–distance curve analysis was performed using JPK data processing software, providing metrics on interaction probability, nature of the bond (bent or tether), binding strength, unbinding position and Young’s Modulus. Characterization of bent- and tether-bonds was performed based on the slope of the force–distance curve immediately before rupture. A bond was classified as bent when the slope was < −60 µN/m, whereas a tether bond was defined by a slope > −60 µN/m, as previously described [19].

### STED/AFM

STED/AFM experiments were done as described previously [15]. Briefly, the original STED microscope stage was replaced with an isolated Life Science stage equipped with a sample holder designed for inverted optical microscopes (Bruker Nano, Berlin, Germany). The NanoWizard 4 AFM scan head was mounted above this setup. Tip calibration was done as described in the AFM section. Next, the stained and living MEKs were positioned on the stage. Precise alignment of the AFM tip over the region of interest was achieved using the standard DirectOverlay™ protocol in the SPM software (JPK Instruments). DirectOverlay™ optically calibrates the AFM scan field with the optical microscope image by moving the AFM tip to nine predefined positions. At each of these positions, the optical microscope acquires an image (scan area: 30 × 30 µm; resolution: 1024 × 1024 pixels). The AFM software then analyses these images to identify the tip’s position, enabling correction for optical distortions and image artifacts automatically. After alignment and calibration, STED images were acquired, followed by AFM topography imaging. The resulting datasets were superimposed within the SPM software, and adhesion force measurements were subsequently performed.

### Recombinant E-Cad-Fc construct

The recombinant human E-Cad-Fc construct was commercially purchased from BioLegend (San Diego, CA, USA). To verify the specificity of the measured E-Cadherin interactions, we applied an inhibitory anti-E-Cad antibody (sc-59778 (DECMA-1), Santa Cruz, epitope binding site is the extracellular domain 1 of E-Cadherin). The antibody was applied to the cell monolayer for 1 hour, followed by three times washing with medium before repetition of force spectroscopy measurements.

### Generation of pSnapf-mE-Cad construct

Complementary DNA (cDNA) was generated from a total RNA, derived from wild type (WT) MEK cells, through reverse transcription. Full-length cDNA of mouse E-cad was obtained by PCR amplification using as a template MEK-derived cDNA with following primers:

**Table:** 

Name of the primer	Sequence 5’->3’
AscI-mE-Cad-FW	TGAATCAGGCGCGCCGCCACCATGGGAGCCCGGTGCCGCA
AgeI-mE-Cad-REV	AATCTAACCGGTGTCGTCCTCACCACCGCCGTACATG

Within the forward primer (AscI-mE-Cad-FW), AscI restriction site was introduced. Similarly, the reverse primer (AgeI-mE-Cad-REV) carried in an AgeI restriction site. Both restrictions sites were used to insert the sticky-ended PCR product into linearized in advance pSNAPf vector (New England Biolabs, N9183S). The desired insert was fused upstream from SNAP-tag. Further restriction and sequencing analyses verified the construct.

### Data processing and statistics

For statistical significance between the two groups, a two-tailed Student’s T-test was applied. For comparison of more than two groups, analysis of variance (one-way ANOVA) followed by Bonferroni post hoc test, was performed. Error bars represent standard deviation. Results were considered as statistically significant when the *p*-values were equal or below 0.05. Each N represents a biological replicate (independent cell seedings from cultured cells with different passage numbers). Figures were prepared with GraphPad Prism 8.

## Results

### E-Cad is present at the leading edge of migrating keratinocytes

**Figure 1.**
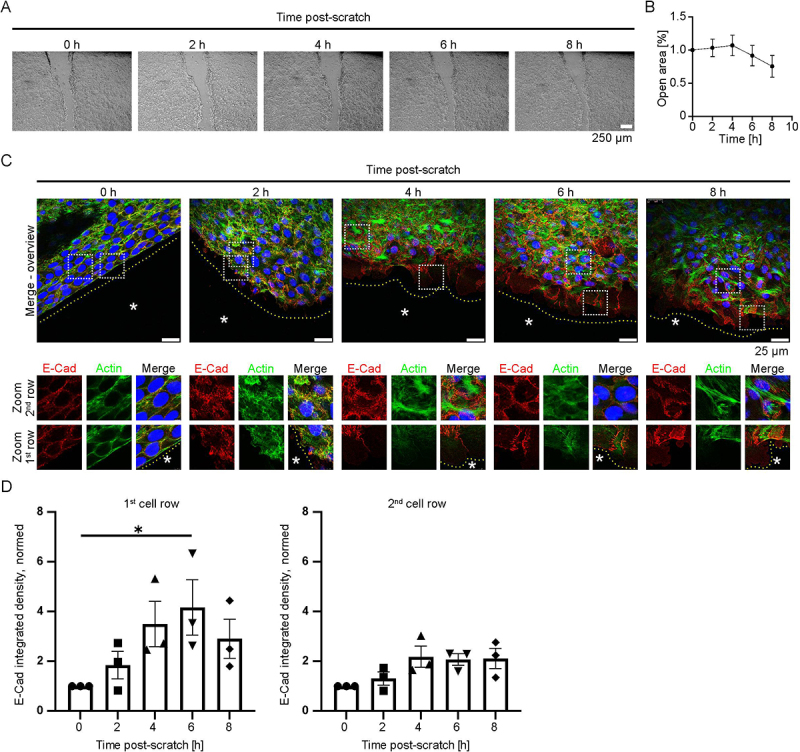
Scratch wound assay reveals elevated expression of E-Cad at the leading edge. (A) MEK were scratched with a pipette tip 24 h after the switch to high Ca^2+^ medium. Images were acquired at 0, 2, 4, 6, and 8 h post-scratch. Each single point depicts the mean of *N* = 5, each N represents a biological replicate, error bars represent the standard deviation. (B) The cell-free area was measured over the 8 h period following scratch. (C) E-Cad (red), F-Actin (green) and nuclei (blue) are visualized 0, 2, 4, 6, and 8 h after scratch at the wound edge. Zoomed-in images distinguish between the first (1st) and second (2nd) rows of MEK adjacent to the wound. The leading edge of the first cell row is indicated by a yellow dotted line in the merge images. The scratched cell-free area is denoted by .* (D) Quantification of E-Cad fluorescence intensity in the 1st and 2nd cell rows at 0, 2, 4, 6, and 8 h post-scratch. *N* = 3; from each N (biological replicate) a representative scratch wound area was imaged; from the overview image the overall fluorescence intensity of the first and second cell row was measured; error bars represent the standard deviation, *p < .05.

To assess the expression of E-Cad during cell migration, we first performed scratch wound assays using MEK (WT) and monitored wound closure over an 8 h period. The scratch was done with a sterile pipette tip, and closure of the wound area was imaged at defined time points for up to 8 h (Figure 1(A)). The quantification revealed that the scratch was reduced to approximately 60% of its initial size after 6- and 8-h post-scratch (Figure 1(B)). Subsequently, we performed immunofluorescence staining for E-Cad and F-actin, as the latter was visualized with Alexa phalloidin. Images were taken at 0, 2, 4, 6, and 8 h post-scratch, with higher magnification views at the wound edge as well as in the following cells (Figure 1(C)). We distinguished between the first and second row of cells next to the wound and quantified E-Cad fluorescence intensity. In the first row, we observed a significant increase in E-Cad intensity 6 h after wounding, with a general upward trend detectable from earlier time points (Figure 1(D)). In the second row, E-Cad intensity also showed an increase from 2 h onward, although these changes did not reach statistical significance (Figure 1(D)). To summarize, we found that E-Cad is present at the leading edge, representing the first cell row of migrating cells 6 h post-scratch. To assess whether MEK at the wound edge exhibit features of epithelial plasticity, we performed immunostaining for the EMT-associated N-Cad marker (Figure S1 A). Immunostaining showed unchanged N-Cad levels and localization in migrating cells compared to cells in the intact monolayer, consistent with a partial EMT-like state.

### E-Cadherin binding properties investigated with the STED/AFM-SMFS

**Figure 2.**
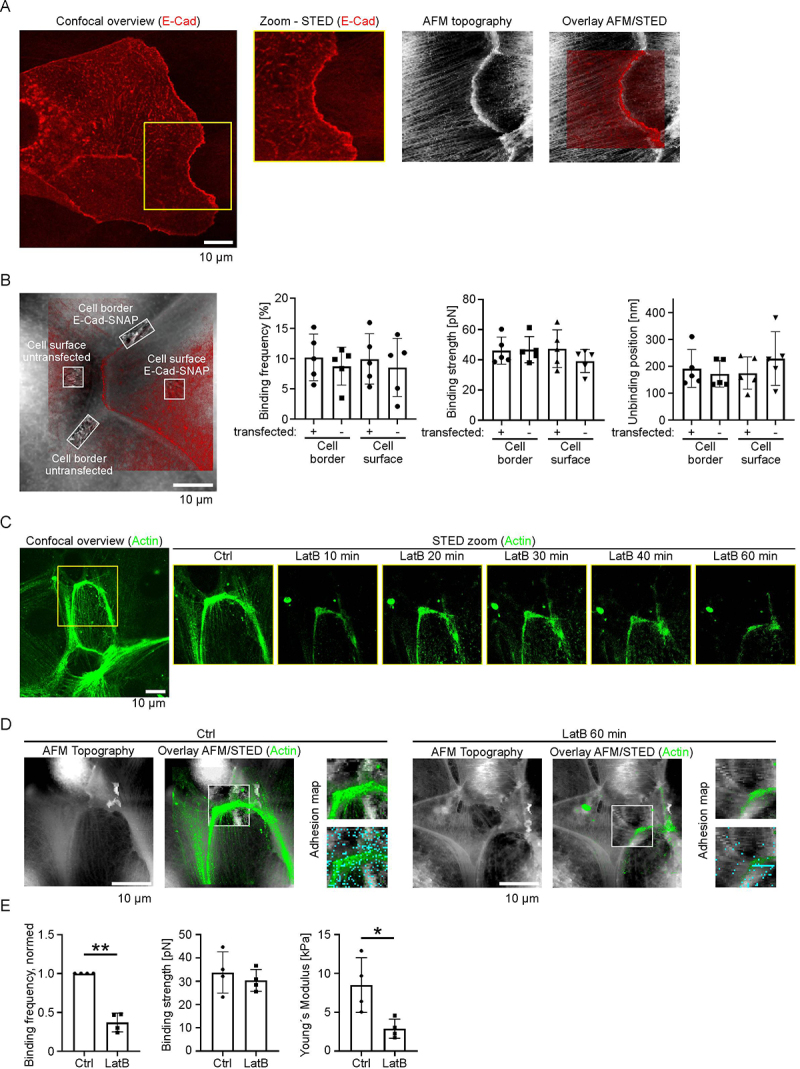
Investigation of E-Cad interactions by STED/AFM. (A) E-Cad-SNAP construct was overexpressed in MEK and labeled with SNAP-CELL-TMR-STAR dye. Overlay images demonstrate that the topographical mapping overlaps the E-cad-SNAP signal. (B) Binding probabilities were measured between an E-Cad-coated AFM tip and distinct cellular regions, including cell borders and cell surfaces, comparing cells with and without E-Cad overexpression. Quantification was done with 5 individual STED/AFM experiments, each consisting of two cell borders and surface areas for transfected and non-E-Cad-transfected cells. (C) Confocal imaging of F-actin labeled with Sir-actin following treatment with LatB for up to 1 h. (D) STED/AFM imaging of MEK labeled with Sir-actin. Cyan dots indicate E-Cad binding events. (E) Quantitative analysis of E-Cad binding events comparing untreated control cells to cells treated with LatB for 1 h; *N* = 4; within each N (representing a biological replicate) one adhesion map consisting of 1600 individual force–distance curves was measured; *p < .05, **p < .01.

Before investigating the E-Cad binding properties in migrating MEK, we aim to measure the basal E-Cad binding properties in confluent MEK. Therefore, we applied the combined STED/AFM-SMFS technique to probe-specific E-Cad interactions at the cellular surface and at cell–cell contact sites. The principal STED/AFM setup is shown in previous publications [14,15]. The AFM tip was functionalized with recombinant E-Cad protein. This technique allowed us to correlate E-Cad localization with AFM-based measurements of E-Cad-mediated adhesion in regions containing E-Cad-SNAP-tagged or endogenous protein. For this purpose, we transiently transfected MEK cells with a pSNAPf-m-E-Cad construct. Prior to STED/AFM-SMFS experiments, cells were labeled with SNAP-CELL-TMR-STAR, enabling specific visualization of the tagged E-Cad protein. Importantly, non-stable E-Cad-SNAP expression was used to visualize the E-Cad localization. The AFM force spectroscopy measurements investigate E-Cad interactions independently of E-Cad overexpression. The E-Cad-SNAP constructs localized predominantly to cell–cell contact sites (Figure 2(A)). High resolution STED imaging revealed a linear pattern of E-Cad staining at contact sites. Correspondingly, AFM topography imaging identified these cell–cell contact sites as elevated regions with raised incoming filaments. The STED signal for E-Cad aligned well with the topographical features observed with the AFM, confirming that the fluorescently labeled protein accumulates at the cell–cell contact (Figure 2(A)). To investigate potential spatial variations, we performed SMFS measurements at cell–cell contact sites (cell borders) with those from regions above the nucleus (cell surface) (Figure 2(B)). Analysis of binding frequency, binding strength, and unbinding position revealed no significant differences between areas with overexpressed and non-overexpressed E-Cad, independent of the cellular area (Figure 2(B)). Across all conditions, the binding frequency remained around 10%, indicating consistent interaction probability. The binding strength was slightly above 40 pN, and the unbinding position was slightly below 200 nm, independent of localization or expression level (Figure 2(B)). The observed findings are in close agreement with previously reported values for specific cell-free homophilic E-Cad interactions measured using the similarly functionalized AFM cantilevers [19]. These findings suggest that E-Cad maintains stable binding characteristics regardless of its local abundance or subcellular distribution.

### E-Cad binding properties depend on the actin cytoskeleton

To assess whether E-Cad binding is influenced by the actin cytoskeleton, we disrupted actin polymerization by using LatB, an inhibitor of actin polymerization. In these experiments, cells were not transfected with E-Cad-SNAP. Instead, SiR-Actin was used to visualize the actin cytoskeleton as a structural reference for STED/AFM measurements. Cells were treated with LatB for 60 min. In parallel live-cell STED imaging revealed a continuous reduction of actin signal intensity within 1 h of LatB treatment, confirming the effective disruption of the cytoskeleton (Figure 2(C)). Despite this, AFM topography imaging demonstrated that overall cellular morphology remained intact in the MEK cells, although STED imaging showed a strong loss of filamentous and cortical actin (Figure 2(D)). SMFS measurements of E-Cad after 1 h of LatB treatment revealed a drastic reduction in binding frequency, indicating E-Cad binding is strongly dependent on the integrity of the actin cytoskeleton. Notably, the binding strength of the remaining E-Cad interactions was unaltered. Furthermore, LatB treatment led to a significant decrease in cellular stiffness, as indicated by a reduction in the Young’s modulus (Figure 2(E)). This supports the conclusion that actin polymerization is crucial not only for E-Cad-mediated adhesion but also for maintaining overall mechanical integrity of the cell.

### E-Cad binding properties at the leading edge of migrating keratinocytes

**Figure 3.**
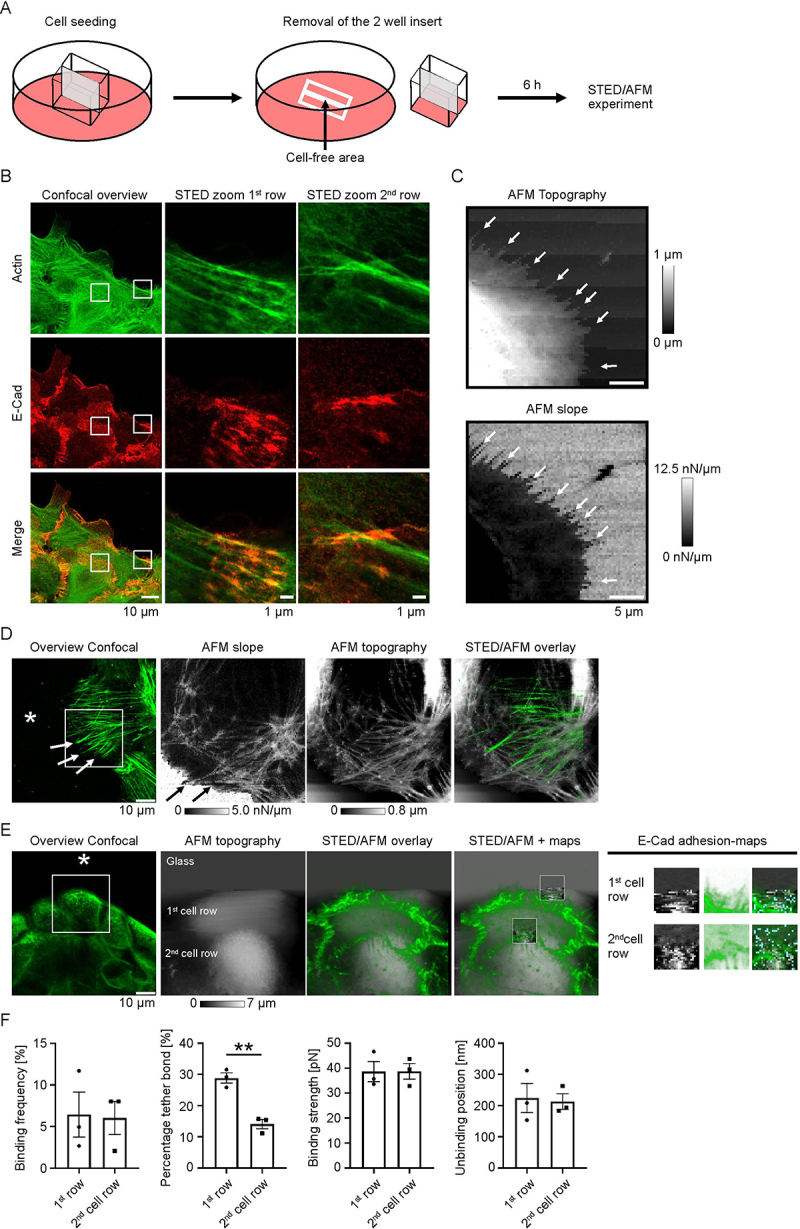
STED/AFM measurements on migrating keratinocytes. (A) Schematic illustration of the experimental setup. A two-well cell culture insert was placed on the coverslip within a 6-well plate. Removal of the insert led to 500 µm cell-free central gap, allowing keratinocyte migration. (B) STED imaging of migrating MEK 6 h post-scratch. Staining was performed for E-Cad and actin cytoskeleton. Image shows representative of *N* = 3, representing biological replicates from independent cell seedings. (C) AFM topography and slope imaging of migrating MEK cells. White arrows indicate the same cellular protrusions, appearing as elevated regions in topography and softer structures in slope imaging, highlighting complementary mechanical information. (D) STED/AFM imaging at the leading edge of migrating MEK. Actin is labeled with sir-actin. The cell-free area is indicated by * and cellular actin protrusions of migrating cells are shown by white and black arrows. (E) STED/AFM imaging at the leading edge of MEK. E-Cad binding measurements were performed at the leading cell (1st row) and at the first cell-cell contact (2nd row). The AFM tip was functionalized with recombinant E-Cad protein. The scratched cell-free area is indicated by .* The measurements conducted at the cell-free area correspond to cover slip (indicated as glass in d). (F) Quantitative analysis of the E-Cad binding events where interactions at the leading edge (1st cell row) were compared with those at the first cell-cell contact (2nd cell row); *N* = 3, each N representing biological replicates from independent cell seedings; **p < .01.

To directly assess the binding properties of E-Cad in migrating non-transfected MEK, we employed the STED/AFM approach. Therefore, actin labeling with SiR-Actin served to guide correlative STED/AFM measurements at the leading edge. To create a defined wound area for migration, a culture-insert 2 well dish was positioned in the center of the AFM coverslip during cell seeding. The placeholder was removed 6 h prior to the STED/AFM experiment, allowing sufficient time for cells to migrate into the 500 µm gap and express E-Cad at the leading edge (Figure 3(A)). STED imaging revealed the presence of E-Cad along actin filaments within the first and second row of migrating cells (Figure 3(B)). AFM measurements of migrating cells demonstrated that topography and slope imaging provide complementary information. While AFM topography visualized cellular protrusions as elevated regions, AFM slope imaging identified these protrusions as mechanically softer structures compared to the glass coverslip. Thus, both imaging channels capture distinct but complementary aspects of MEK cell migration (Figure 3(C)). Next, we imaged the actin cytoskeleton at the leading edge by applying SiR-Actin staining and probed E-Cad interactions at the single-molecule level. For this, AFM tips were functionalized with recombinant E-Cad to enable specific binding interactions. Confocal imaging at the wound edge revealed the presence of actin fibers oriented toward the gap, consistent with AFM topography and slope measurements (Figure 3(C,D)). Corresponding AFM measurements at the same location showed increased rigidity in cellular regions with underlying cytoskeletal filaments, including actin, which are orientated toward the adjacent cell-free space. These stiff cellular structures correlated with elevated topographical features seen in the AFM topography and with actin-protrusions clearly visualized in the STED/AFM overlay (Figure 3(D)). These findings indicate that migrating cells can be investigated by the STED/AFM technique.

Finally, we performed E-Cad adhesion measurements using the STED/AFM technique at distinct regions of migrating non-transfected MEK, namely the first cell row and the second cell row, which already assembled cell–cell contacts (Figure 3(E)). We selected cells at the leading edge of the wound which displayed polarized morphology and actin-rich protrusions toward the cell-free area. These cells were therefore considered to represent the migrating population. To verify the specificity of the E-Cad interactions, an inhibitory anti-E-Cad antibody (Ab) was applied for 1 h following control measurements. Quantitative analysis revealed a reduction in binding frequency after Ab treatment, confirming that the detected interactions were mainly specific homophilic E-Cad interactions (Figure S1 B and C). E-Cad binding events were detected already at the cellular surface of the first cell row, indicating that E-Cad proteins are present for interactions at the leading edge. Binding frequency was not increased in the second cell row directly behind. For calculation of the binding frequency, we subtracted the cell-free area. Interestingly, the proportion of tether bonds, which are indication for cadherins weakly anchored to the cytoskeleton [15,], was significantly higher at the leading edge compared to the second cell row. In contrast, the binding strength and unbinding position did not alter between the two examined regions and remained consistent with the above presented measurements (Figure 3(F) and see Figure 2(B)), supporting the conclusion that E-Cad binding properties are conserved across migrating cell rows, despite the local differences in cytoskeletal anchorage.

## Discussion

In this study, we demonstrated that STED/AFM is a versatile technique for investigating keratinocyte migration, enabling both the measurement of cellular mechanical properties and single-molecule force spectroscopy of cadherins. We found that E-Cad is expressed at the leading edge of migrating MEKs and in this localization exhibits binding properties similar to those noticed in confluent cells. Using the combined STED/AFM approach, we further demonstrated that E-Cad binding depends on an intact actin network, which is itself essential for maintaining cellular stiffness. Furthermore, we showed that STED/AFM can be effectively applied to study the biophysical characteristics of migrating cells as well as the single molecule binding properties of their junctional molecules.

Scratch wound assays are widely used across various scientific fields, such as cell biology, cancer research, dermatology, and regenerative medicine, to study cell migration, cell–cell interactions, proliferation, and tissue regeneration [22–25]. The assay evaluates the rate of wound closure, which can be compared under different treatment conditions to assess the efficacy of therapeutic compounds. It is a relatively simple, cost-effective and easy-to-perform method for investigating wound healing and cellular migration. The technique mimics the *in vivo* process of cell migration during tissue repair. The main steps include creating a wound, typically by scratching a confluent cell monolayer with a pipette tip, capturing images at regular time intervals during the wound closure process, and analysing these images to quantify the cell migration rate [23]. Scratch wound assays have also been applied in the desmosomal field, where it was shown that desmoglein 3 regulates the transition to a migratory keratinocyte phenotype through the inhibition of the p38MAPK pathway [26]. Furthermore, it was shown that wounding leads to a transition of hyperadhesive to non-hyperadhesive desmosomes, via relocation of protein kinase C α towards the desmosomal plaque, resulting in a loss of desmosomal adhesion and thus promoting towards a migratory state of the keratinocytes [27,28]. We note that scratch wound assays inherently combine collective cell migration and cell proliferation. Here, our analysis is restricted to early time points (≤8 h post-scratch), a time phase which is considered to be dominated by cell migration in epithelial monolayers, whereas proliferation contributions become more relevant at later stages of wound healing [29]. Therefore, cells at the wound edge which display a polarized morphology and possess actin-rich protrusions toward the cell-free area were defined as migrating cells [30,31].

The mechanical properties and cadherin-binding properties of migrating keratinocytes have not been investigated before. Here, we demonstrate that the STED/AFM technique can be successfully applied to measure biophysical properties of migrating keratinocytes. We found that E-Cad expression was enhanced at cell–cell junctions at the leading edge 6 h after scratch. We investigated the E-Cad binding properties using an E-Cad-functionalized AFM tip in MEK cells, with overexpressed E-Cad-SNAP only where visualization of E-Cad localization was required. The binding probability was approximately 10%, consistent with cadherin interaction probabilities previously reported for living MEKs [18,19,32]. The measured binding strength was slightly above 40 pN, which is within the range described in earlier studies [33–35]. The agreement of these parameters with published single-molecule E-Cad force spectroscopy data further supports the functional integrity of the tip-anchored E-Cad extracellular domains. However, we did not determine the relative abundance of exogenously expressed E-Cad to endogenous E-Cad. As a result, AFM measurements cannot discriminate between the adhesive interactions involving endogenous versus exogenous E-Cad. The data therefore reflects the overall E-Cad-mediated adhesion at the cell surface and cell–cell contacts and does not distinguish its molecular origin. Importantly, non-transfected cells neighboring the transfected cell exhibited similar E-Cad binding properties, showing that endogenous E-Cad binding properties do not differ from overexpressed E-Cad binding properties. By applying the actin polymerization inhibitor LatB, we demonstrated that the frequency of E-Cad binding was drastically reduced, demonstrating that an intact actin cytoskeleton is functionally required for maintaining E-Cad-mediated adhesion under these conditions. Because actin polymerization was globally disrupted, these experiments do not distinguish between direct and indirect effects of E-Cad binding but instead demonstrate a functional dependence of E-Cad-mediated adhesion on cytoskeletal integrity. In addition, we observed that disruption of the actin cytoskeletal network resulted in a loss of cellular stiffness, and this observation was consistent with previous findings [36,37]. Decrease in stiffness, reflected by a significant decrease in the Young’s modulus, indicates that actin filaments are key contributors to the mechanical integrity of cells [38].

Using the hybrid STED/AFM technique on migrating keratinocytes, cellular protrusions were visualized by live-cell STED imaging, which resemble more rigid and elevated structures observed in the AFM slope and height channel. Interactions between the E-Cad-coated tip and cellular binding partners revealed that molecules at the leading edge exhibit a higher percentage of tether bonds compared to those at the second cell row directly behind. This suggests that the binding partners at the leading edge are likely not firmly anchored and serve more as sensory, extra-junctional cadherins rather than primarily adhesive molecule [39].

This study demonstrates the feasibility of combining STED/AFM with single molecule force spectroscopy measurements to investigate migrating cells, potentially bridging the gap between biophysical characterization and dynamic migration assays. This approach offers valuable insight into the mechanobiology of wound healing, an area that current methods can only partially address. Furthermore, the high-resolution STED imaging enables the correlation of mechanical measurements with cytoskeletal dynamics and protein localization within the migrating cells. In the context of cell–cell adhesion biology, this technique can be used to quantify adhesion forces at the molecular level and to investigate the dynamic remodeling of junctional complexes during migration. Although AFM measurements can also be combined with confocal microscopy, the advantage of the STED/AFM setup lies within the high-resolution imaging of cellular structures. STED imaging allows precise localization of AFM interaction sites, relative to cellular structures of the leading edge or actin-rich protrusions, which cannot be reliably distinguished at confocal resolution. As demonstrated in this study, we measured the binding properties of E-Cad at the leading edge and compared them to those at stable cell–cell contacts. We found that both E-Cad pools exhibit similar binding properties, indicating that E-Cad molecules at the leading edge are functional and capable of mediating adhesion. For cancer research, this technique might offer a platform to study early mechanical changes associated with EMT and to identify biophysical properties of invasive cell behavior; for example, downregulation of E-Cad. In our study, the MKEs involved in wound healing are not thought to undergo a complete EMT, but instead adopt an EMT-like state, where they possess epithelial characteristics and in parallel acquire migratory properties. To distinguish these two states, the dynamic processes which occur in keratinocytes were proposed to be named epithelial–mesenchymal plasticity [11]. Here, we focus on this epithelial plasticity and its impact on cadherin-mediated adhesion. Thus, in this study, we introduce a method to investigate the biophysical properties of single molecules during keratinocyte adhesion, which could lead to a better understanding of migration processes in tissue repair and wound healing.

## Supplementary Material

Supplementary Information.docx

## Data Availability

The data that support the findings of this study are available from the corresponding author upon reasonable request.
