# Supplementary material for: STED/AFM as a tool to investigate mechanical and adhesive properties of migrating keratinocytes
Source: Cell Adh Migr. 2026 Apr 15;20(1):2657675. doi: 10.1080/19336918.2026.2657675 (PMC13089918; doi:10.1080/19336918.2026.2657675)
Supplement: Supplementary Information.docx [file KCAM_A_2657675_SM1521.docx]

**Supplemental Materials**

**STED/AFM as a tool to investigate mechanical and adhesive properties of migrating keratinocytes**

Mariya Y. Radeva, Jens Waschke and Michael Fuchs

Chair of Vegetative Anatomy, Institute of Anatomy, Faculty of Medicine, LMU Munich

Address for correspondence:

Dr. Michael Fuchs

Chair of Vegetative Anatomy, Institute of Anatomy, Faculty of Medicine, LMU Munich

Pettenkoferstr. 11, 80336 Munich, Germany.

Email: [M.Fuchs@med.uni-muenchen.de](mailto:M.Fuchs@med.uni-muenchen.de) Phone: +49-89-2180-72614

**Figure S1**

**
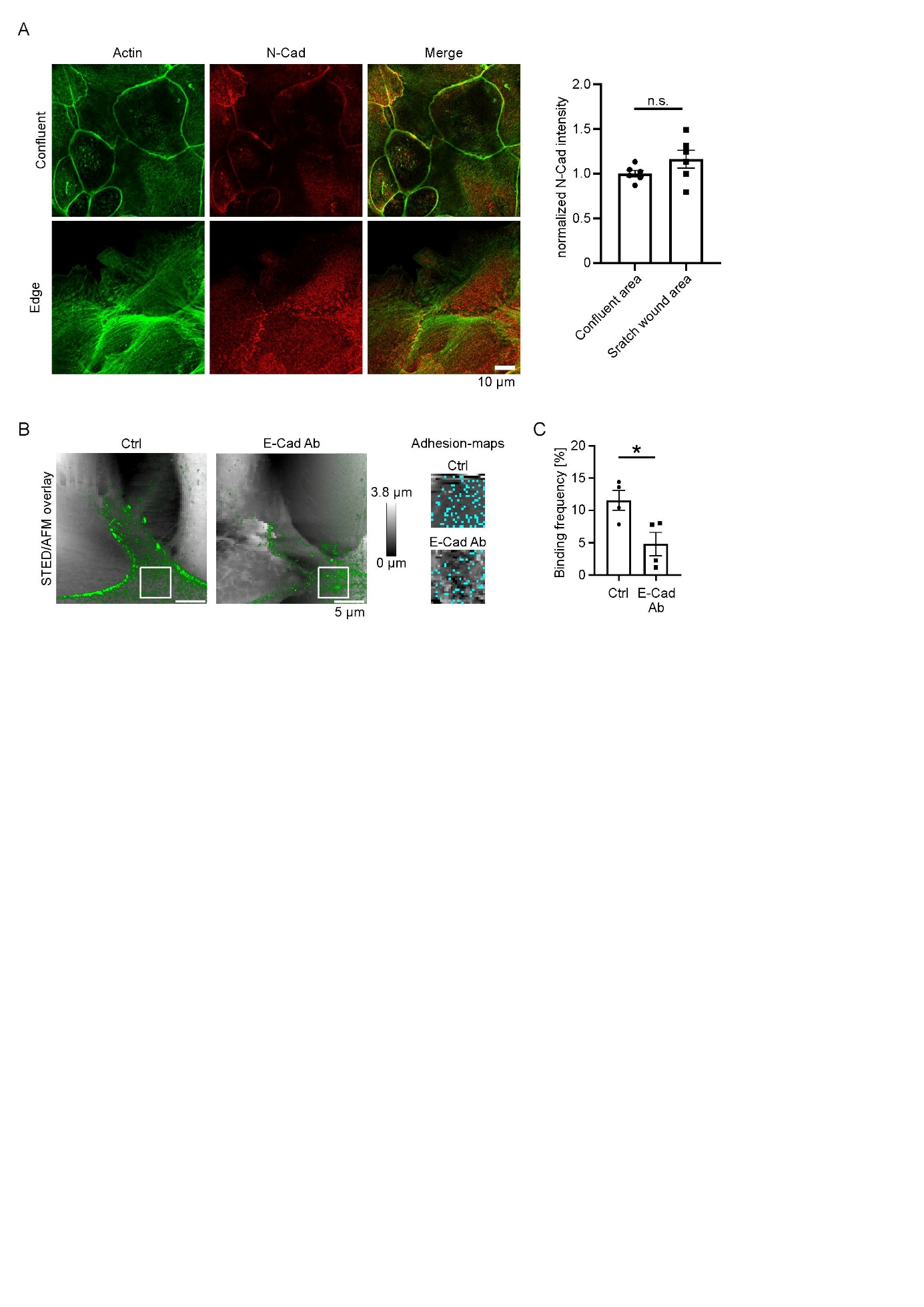
**

**Figure legends**

**Figure S1: A:** Immunofluorescence staining for N-Cad and actin comparing migratory MEK cells at the wound edge with cells in confluent regions. Quantification of N-Cad fluorescence intensity from N=6 biological replicates.  **B:** Combined STED/AFM imaging of MEK cells with F-actin labeled using Sir-actin. Force-spectroscopy measurements were performed using an E-Cad-functionalized AFM tip before and after treatment with an inhibitory E-Cad antibody for 1 h, followed by extensive washing. Cyan dots in the adhesion maps indicate E-Cad binding events between the functionalized tip and the cell surface. **C:** Quantitative of E-Cad binding events comparing control cells and cells incubated with the inhibitory E-Cad antibody for 1 h followed by antibody removal and washing; N=4; *p<0.05.
